# Supplementary material for: Ethnicity and risk of death in patients hospitalised for COVID-19 infection in the UK: an observational cohort study in an urban catchment area
Source: BMJ Open Respir Res. 2020 Sep 1;7(1):e000644. doi: 10.1136/bmjresp-2020-000644 (PMC7467523; doi:10.1136/bmjresp-2020-000644)
Supplement: Supplementary data [file bmjresp-2020-000644supp001.pdf]

Ethnicity and risk of death in patients hospitalised for COVID-19 infection: an observational cohort study in an urban catchment area

E Sapey, S Gallier, C Mainey, P Nightingale, D McNulty, H Crothers, F Evison, K Reeves, D Pagano, A.K Denniston, K. Nirantharakumar, P Diggle, S Ball on behalf of all clinicians at University Hospitals Birmingham NHS Foundation Trust

Online Supplement

Supplementary Table S1. Standardised Admission rates and Standardised Mortality Rates for admitted patients with confirmed COVID-19

Ethnicity	Standardised Admission Rate (95% confidence interval)		Standardised Mortality Rate (95% confidence interval)	
	Female	Male	Female	Male
White	0.84 (0.78 - 0.91)	0.87 (0.81 - 0.93)	0.89 (0.78 - 1.01)	0.90 (0.81 - 0.99)
Asian	1.74 (1.49 - 2.02)	1.63 (1.44 - 1.85)	1.93 (1.42 - 2.58)	1.73 (1.38 - 2.16)
Black	1.21 (0.93 - 1.55)	1.03 (0.82 - 1.29)	1.03 (0.57 - 1.72)	1.08 (0.72 - 1.55)
Mixed	1.03 (0.50 - 1.89)	0.72 (0.35 - 1.33)	2.86 (0.91 - 6.89)	1.24 (0.39 - 3.00)
Other	2.79 (1.92 - 3.93)	1.95 (1.40 - 2.67)	0.86 (0.14 - 2.85)	0.88 (0.32 - 1.95)

Legend: Admission rates were compared to the 2011 census data for Birmingham and Solihull.

Figure S1 of the online supplement. Modified CONSORT diagram of patients

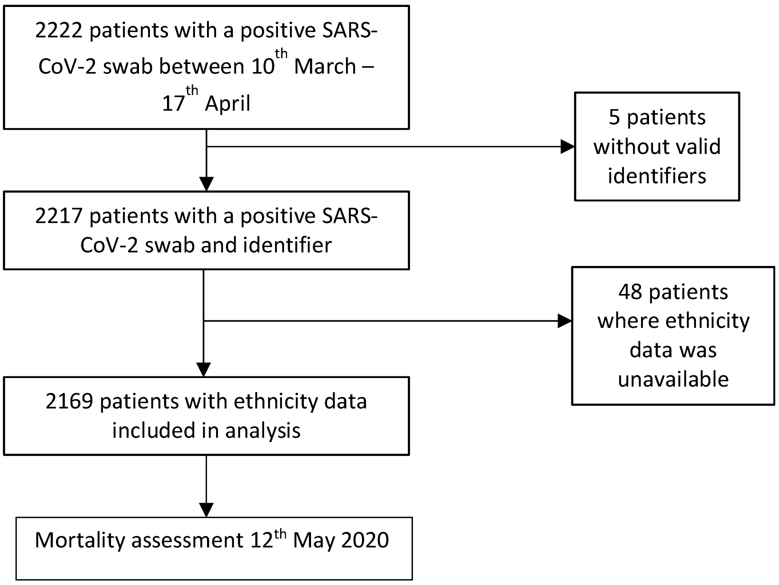

Legend. A modified CONSORT diagram to demonstrate where patients could not be included in analysis and the reasons for this. In total 53 patients (reflecting 2.3% of the SARS-CoV swab positive patients) could not be included in the ethnicity analysis

Figure S2 of the online supplement. The proportion of patients being diagnosed with COVID-19

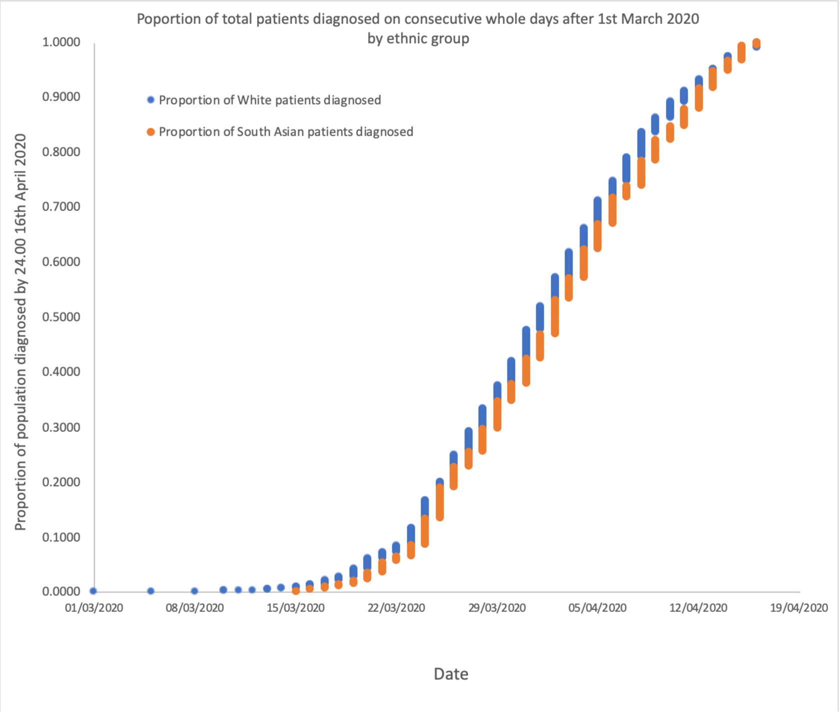

Legend. A figure to show the proportion of patients from White or South Asian ethnicity being diagnosed with COVID-19 (swab confirmed) at UHB by date. There were no differences in diagnostic rates across the population.
